# Practical Medicine

**Published:** 1878-12

**Authors:** 


*Collaborators:*

DR. H. GRADLE, DR. L. W. CASE, DR. R. PARK,
DR. R. TILLEY, DR. D. R. BROWER.

### PRACTICAL MEDICINE.

THE ETIOLOGY OF TYPHOID FEVER. By Prof. Huguenin. (*Corresp. Blatt. f. schweiz. Aerzte.*, 1878, Aug. 1.)

On the 30th of June, 1878, the festival of the singing societies of the district was celebrated in Kloten (Canton Zürich, Switzerland), with a very large attendance. The meals were served at the hotel "Zum Wilden Mann," where about 800 to 900 pounds of viands, partly roast veal, partly sausages, were consumed. Since a large portion of the viands was found somewhat unpalatable, it was distributed gratuitously. It is not yet known where all the meat was obtained, but so much is certain, that at least one calf used was sick before it was slaughtered. The liver and brain of that calf had been consumed in the village Seebach, and had caused illness of the consumers. The rest of the carcass was sent to the hotel at Kloten.

This veal turned out to be infectious. The second day after the festival a number of inhabitants of the neighboring villages complained of nausea, anorexia, headache, fever, abdominal pains and meteorism. The first cases seem to have been the least severe; at least, a number recovered after a few days. More commenced on the third and fourth days, but most from the fifth to ninth day. The first 40 to 50 instances proved the veal to be the source of the infection. Pork had also been consumed that day, but no case could be traced to it. A large number of persons had visited the celebration, but only drank wine, and none of them became infected. Others had partaken of the water without evil consequences. Many of the patients, on the other hand, declared that they had not even tasted water the entire day. Those who had partaken of the three different preparations of the meat were most seriously stricken, while the less severe cases had eaten only one kind of meat. Many, who had induced vomiting by excessive drinking, remained healthy.

The disease caused by this meat was typhoid fever (abdominal typhus). The fever followed the typical course in most cases. But all observers noticed the tendency to mental troubles, furious delirium, with a rather low febrile movement, and rapid favorable course. The delirium continued, in many instances, after antipyretic treatment had removed the fever. Another characteristic feature of the epidemic was enormous tumefaction of the spleen.

In four fatal cases, the autopsy has revealed all characteristic lesions of typhoid fever.

As a noticeable event, Prof. Huguenin adds, that several instances of genuine typhoid fever have occurred in calves in the barns of some of the patients.

The Nature of Sciatica. M. Fernet. (*Lyon Médical.*)

The author has pursued his researches which go to prove that primitive spontaneous sciatica, ordinarily due to a local chilling, should not be considered as a simple neuralgia, that is to say, as an affection without appreciable anatomical lesion, but rather as a veritable neuritis. Exposing the results obtained in the *Archives de Médecine*, M. Fernet remarked that he relied chiefly for the establisment of his opinion upon three clinical characters: the direct examination of the nerve by palpation, the frequent existence of trophic troubles, and the course of the disease. Recently, in the case of a man in his wards affected with sciatica, he had occasion to examine *post mortem*, the state of the diseased nerve, and he found a manifest increase of volume, as well as a very marked injection of the nerve, but this augmentation of volume may be readily perceived during life. Here are the directions which M. Fernet furnishes upon this subject: The patient lying upon the back with the thighs slightly flexed upon the pelvis, and the legs upon the thighs, is directed to keep his lower limbs at perfect rest and to make no effort. You then explore the sciatic nerves with the fingers, which are pushed rather deeply into the popliteal space at first, then proceeding progressively upwards to the sciatic notch. The fingers being well engaged in the depth of this space, their palm or face turned towards the outer aspect of the thigh, and their extremities being occasionally carried from within outwards, the sciatic nerve is very distinctly felt under the form of a cord, and when this is firmly pressed you are apprised of the fact by the sensation which the patient experiences, a sensation only unpleasant on the sound side, but painful and accompanied with tinglings in the leg and foot of the affected side. The palpation is only really difficult in very fat subjects, or when the sciatic is very painful: in this latter case, pressure on the nerve is intolerable, and provokes reflex contractions which prevent the exploration.

By this proceeding, carefully applied, there are frequently found very notable differences of volume between the healthy and the diseased side, the nerve of the affected side appearing larger than that of the sound, a difference of consistence, the nerve of the affected side being harder than that of the sound side; a difference of form, the nerve of the affected side forming a cylindrical cord which pressure does not modify, whilst the nerve of the sound side appears to allow itself to be flattened out and even dissociated. The lesion which is thus discovered may, moreover, be confined to certain points of the nerve.

The nature of the pain, which is continuous, persistent at first, dull, and gradually intensified, limited to the nerve trunk, or even to a part of the trunk without constant peripheral radiations is also, according to M. Fernet, a further proof of the existence of a nerve-inflammation. The defects of nutrition, which, as M. Charcot has shown, are dependent upon inflammatory lesions of the nervous system, are not wanting here. There is often muscular atrophy of the leg and thigh, easily appreciated upon measurement.

At the same time with the muscular atrophy there is a thickening of the subcutaneous cellular tissue by a deposit of fat; these two states appear to be in habitual connection with one another; and in order to appreciate this adiposis, it suffices to take up at symmetrical points on the two thighs or the two legs, a fold of the skin, and to pinch it moderately between the thumb and finger; you can then very readily recognize the greater thickness which exists on the diseased side: this thickening may be sufficiently great to mark the atrophy and to give to the limb a rounded form often noted in sciatica.

Zona, which is always an index of nerve inflammation, also sometimes appears in sciatica; lastly, the evolution of the disease may also be invoked, as being contrary to the hypothesis of a simple functional trouble devoid of lesion.

In a therapeutic point of view, it results hence, that if it be admitted that primitive, spontaneous sciatica is usually a neuritis, a resolutely antiphogistic medication will be employed against it: absolute rest, leeches, wet cups in the course of the nerve, vesicating strips at the back of the thigh, cauteries, etc., will be the chief means of treatment.

